# Approach for Phased Sequence-Based Genotyping of the Critical Pharmacogene Dihydropyrimidine Dehydrogenase (*DPYD*)

**DOI:** 10.3390/ijms25147599

**Published:** 2024-07-11

**Authors:** Alisa Ambrodji, Angélique Sadlon, Ursula Amstutz, Dennis Hoch, Martin D. Berger, Sara Bastian, Steven M. Offer, Carlo R. Largiadèr

**Affiliations:** 1 Department of Clinical Chemistry, Inselspital, University Hospital of Bern, University of Bern, INO-F, 3010 Bern, Switzerland; alisa.ambrodji@extern.insel.ch (A.A.); angelique.sadlon@insel.ch (A.S.); ursula.amstutz@insel.ch (U.A.); 2 Graduate School for Cellular and Biomedical Sciences, University of Bern, 3012 Bern, Switzerland; 3 Department of Medical Oncology, Inselspital, University Hospital of Bern, 3010 Bern, Switzerland; dennis.hoch@insel.ch (D.H.); martin.berger@insel.ch (M.D.B.); 4 Department of Medical Oncology, Cantonal Hospital Graubünden, 7000 Chur, Switzerland; sara.bastian@ksgr.ch; 5 Department of Pathology, Carver College of Medicine, University of Iowa, Iowa City, IA 52242, USA; steven-offer@uiowa.edu

**Keywords:** pharmacogenomics, *DPYD*, fluoropyrimidines, haplotype, PCR chimera, long-range amplicon, rare variants, compound heterozygous, Oxford Nanopore Technologies sequencing

## Abstract

Pre-treatment genotyping of four well-characterized toxicity risk-variants in the dihydropyrimidine dehydrogenase gene (*DPYD*) has been widely implemented in Europe to prevent serious adverse effects in cancer patients treated with fluoropyrimidines. Current genotyping practices are largely limited to selected commonly studied variants and are unable to determine phasing when more than one variant allele is detected. Recent evidence indicates that common *DPYD* variants modulate the functional impact of deleterious variants in a phase-dependent manner, where a *cis*- or a *trans*-configuration translates into different toxicity risks and dosing recommendations. *DPYD* is a large gene with 23 exons spanning nearly a mega-base of DNA, making it a challenging candidate for full-gene sequencing in the diagnostic setting. Herein, we present a time- and cost-efficient long-read sequencing approach for capturing the complete coding region of *DPYD*. We demonstrate that this method can reliably produce phased genotypes, overcoming a major limitation with current methods. This method was validated using 21 subjects, including two cancer patients, each of whom carried multiple *DPYD* variants. Genotype assignments showed complete concordance with conventional approaches. Furthermore, we demonstrate that the method is robust to technical challenges inherent in long-range sequencing of PCR products, including reference alignment bias and PCR chimerism.

## 1. Introduction

Fluoropyrimidines (FPs), including 5-fluorouracil (5-FU) and its oral prodrug capecitabine, are amongst the most commonly used anticancer drugs and are frequently used to treat solid tumours [1]. Despite their effectiveness, they cause severe adverse events (i.e., toxicity) in 10–40% of patients [2]. Decreased function of dihydropyrimidine dehydrogenase (DPD), which catabolizes approximately 80–85% of administered 5-FU to 5-fluorodihydrouracil (F-UH_2_), is one of the primary risk factors for FP toxicity. Reduced DPD activity increases systemic exposure to 5-FU, which can in turn lead to overexposure to the active cytotoxic metabolites. DPD activity is highly variable in the population, with an estimated 3–8% being partially DPD-deficient, partly attributed to genetic variability in its encoding gene, named *DPYD* [3]. Currently, four well-characterised single nucleotide polymorphisms (SNPs) in *DPYD*, c.1905+1G>A (rs3918290), c.1679T>G (rs55886062), c.2846A>T (rs67376798), and c.1129-5923C>G (rs75017182, c.1236G>A/HapB3) are recommended for genotyping to identify patients with increased FP toxicity risk [4,5]. Carriers of *DPYD* risk variants have 25.6 times higher risk of FP-related death [6]. A recent survey showed that routine pre-treatment targeted genotyping of these four variants has been widely implemented in Europe [7], following the recommendation by the European Medicines Agency (EMA) in 2020 to test for DPD deficiency prior to FP treatment [8].

Tests for the aforementioned variants have been suggested to have limited utility outside of individuals with solely European ancestry since the four variants are comparatively rare in other populations, where additional variants have been suggested to significantly contribute to toxicity risk [9,10,11]. Addressing this additional genetic variability necessitates moving beyond conventionally used targeted genotyping. In line with these observations, sequencing of the entire gene has been proposed to improve pre-treatment testing for FP toxicity risk [12,13]. While the *DPYD* coding sequence is 4.4 kb, the gene’s 23 exons span 950 kb, complicating sequence-based genotyping in DNA.

Recent evidence from our lab demonstrates that the haplotype phasing between multiple *DPYD* variants is a critical determinant for DPD enzyme function [14]. Additionally, current dosing guidelines provide differential recommendations based on carriers heterozygous for two risk variants, depending on if the variants are in *cis*- or *trans*-configurations [15].

Here, we report an Oxford Nanopore Technologies-based method for genotyping and haplotype phasing of the entire coding region of the important pharmacogene *DPYD* that utilizes mRNA as a starting point. We assess the accuracy of this approach relative to conventional genotyping methods and address optimization strategies to mitigate potential bias associated with the sequencing of long-range PCR products.

## 2. Results

### 2.1. Genotype Validation

To assess the suitability of long-range sequencing using the Oxford Nanopore Technologies platform for genotyping *DPYD*, we generated amplification products that span the entire protein coding region of the *DPYD* gene (Supplementary Table S1) using RNA from 21 individuals. We first assessed the concordance with conventional genotyping methods by comparing the Nanopore genotyping results with results from TaqMan genotyping (Supplementary Table S1). A total of 142 genotypes from 21 individuals had been generated using nine validated *DPYD* TaqMan SNP assays prior to this study. These included 37 heterozygous and 105 homozygous wildtype (wt) genotypes. We observed complete concordance between results from Nanopore-based and TaqMan genotyping methods. For these genotypes, thus, no false negative genotype calls were detected. Notably, full-length sequencing yielded 11 additional heterozygous genotypes (see Supplementary Table S1 for details). We also did not record any false positive calls, as all of the 11 sites were *a posteriori* confirmed by TaqMan and Sanger sequencing.

### 2.2. Impact of PCR Conditions on PCR Chimerism

The formation of PCR chimeras is an issue inherent in long-range PCR reactions. Chimeras can arise if incompletely elongated copies act as primers, annealing to the homologous template copy in the following PCR cycles. If the sequence acting as a primer contains a polymorphic site, which is not present in the primed template, the two sites are artificially recombined on the resulting copy [16]. Increasing PCR amplification cycles, template size, and template input have been reported to promote the formation of such artefacts [17,18].

To determine the effect of reaction conditions on the formation of PCR chimeras and true haplotypes, we compared two-loci haplotype frequencies in amplicons of two samples (referred to as C1 and C2) generated with different PCR conditions and input DNA amounts (Supplementary Table S2). The two samples were heterozygous at four and three loci in *DPYD* (C1: c.85T>C, c.1236G>A, c.1627A>G, c.2846A>T, and C2: c.85T>C, c.496A>G, c.1236G>A), respectively. As PCR chimerism will generate additional haplotypes, we use the term compatible haplotype pairs for combinations of two haplotypes that are compatible with the observed diploid genotype. For example, for an individual who is heterozygous at two positions in a gene, there are four different haplotypes possible, consisting of two compatible haplotype pairs, i.e., a pair of true haplotypes and a pair of recombined haplotypes. In both samples, for all nine PCR profiles, we observed all four possible haplotypes when looking at any two variant combinations. This indicates the presence of PCR chimeras in all experimental conditions. In addition, we observed a fifth category of sequences that could not be assigned to any of the four haplotypes. We classified these sequence reads into a single group. This small subset of sequence reads consisted of alternative basecalls at the variant positions, or local misalignment due to insertions or deletions (see Table S2 for more details). 

**Figure 1:**
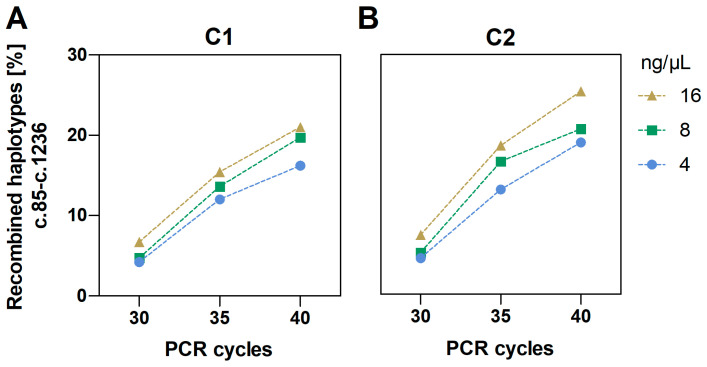
Frequency of the recombined two-locus haplotypes (PCR chimeras) of c.85T>C and c.1236G>A in sample C1 (**A**) and sample C2 (**B**) according to PCR conditions: number of PCR amplification cycles (x-axis) and DNA template input.

We counted the number of aligned reads supporting each of the four possible haplotypes formed. In general, we observed that one particular compatible haplotype pair increased in relative frequency, while the other compatible haplotype pair decreased with less stringent PCR conditions, i.e., higher cycle numbers and template input (see Figure 1 for c.85T>C-c.1236G>A and Supplementary Table S2 for all others). Since less stringent PCR conditions favour the formation of PCR chimeras, we determined the decreasing haplotype pair as being the true phased genotype of the individuals. 

As can be seen in Figure 1 and Supplementary Tables S2 and S3, both higher PCR cycle number and cDNA input significantly contributed (all *p* < 0.001) to the increase in PCR chimeras. For the two variants present in both samples, c.85T>C and c.1236G>A, the true haplotype pair accounted for 90.8% of the aligned sequence reads in C1, and 89.4% in C2, whereas the chimeric haplotypes had frequencies of 4.2% and 4.5% with the most stringent PCR conditions, i.e., lowest cycle number and input. The relative frequency of chimeric reads increased to 6.7% and 7.4% when increasing the input from 4 ng/µL–16 ng/µL, while increasing the cycle number from 30–40 increased the frequency to 16.2% in C1 and 19.0% in C2. The remaining sequences that could not be assigned to any of the four haplotypes, accounted for 4.2–5.4% and 5.1–6.1% of the reads in all conditions in C1 and C2, respectively (Supplementary Table S2). The same pattern was observed for all other two-locus variant-combinations in both samples.

### 2.3. PCR Recombination According to Distance

**Figure 2:**
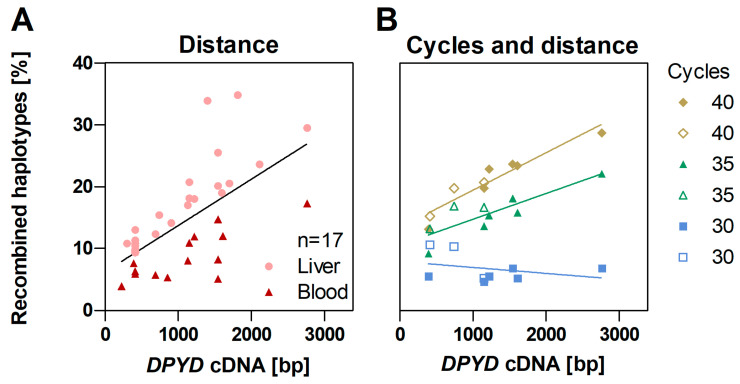
The relative frequency of recombined two-locus haplotypes (PCR chimeras) in relation to distance (base pairs; bp) between variant sites (**A**) in 17 samples, using template input of 8 ng/µL and 35 PCR amplification cycles, and (**B**) in samples C1 (filled symbols) and C2 (empty symbols) at 8 ng/µL input and 30, 35, and 40 PCR amplification cycles.

We next investigated the relationship between PCR-mediated recombination and physical distance between variant sites, which was measured in base pairs between variants. The impact was assessed in 17 of the 21 sequenced individuals, which met the qualification criteria by carrying more than one variant. In addition to the 17 samples, which were prepared using 8 ng/µL template input and 35 amplification cycles, samples C1 and C2 were evaluated under different PCR conditions. Variants located further apart were more likely to be recombined than variants at shorter distances (Figure 2A,B). Furthermore, samples based on RNA extracted from liver tissue recombined at a higher rate than those extracted from blood cells (Figure 2A). This observation is consistent with cDNA template input on PCR recombination, since *DPYD* is highly expressed in liver, with lower expression in blood compartments. The tendency to recombine at a higher rate with increased distance was mainly observed at 35 and 40 amplification cycles, as seen in Figure 2B, when using 8 ng/µL template input. The same trend was seen for 4 ng/µL and 16 ng/µL template input (Supplementary Table S2), indicating that optimization of reactions to minimize both the number of cycles and input DNA while still generating adequate product for sequencing is an effective strategy to limit PCR chimerism.

### 2.4. Reference Bias of Alignments

**Figure 3:**
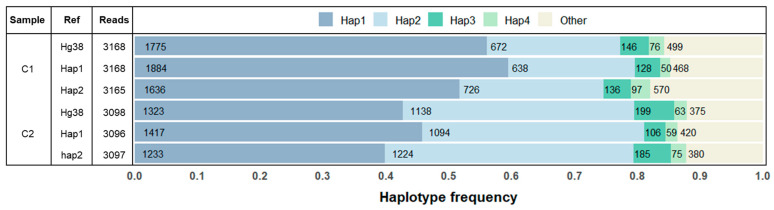
Absolute and relative frequencies of phased haplotypes in samples C1 and C2 generated with 30 PCR amplification cycles and 4 ng/µL input. Reads aligned to reference sequence based on hg38, hap1, and hap2. Hap1–hap4 represent the four most abundant haplotypes. The category “Other” encompasses all remaining haplotypes. Hap: haplotype; ref: reference.

Sequence alignment algorithms favour sequences with closer resemblance to the reference they are mapped to. The presence of variants can therefore skew which sequences are aligned, and which are discarded by the algorithm [19]. To determine the extent to which alignment bias affects results in long-range sequencing, we used samples C1 and C2 with PCR conditions of 30 amplification cycles and 4 ng/µL input. The reads were aligned to the *DPYD* mRNA reference sequence from hg38, and to references specific to the two previously inferred dominant haplotypes for each sample. As expected, substituting the bases in the reference to match the specific haplotypes slightly shifted the mapped sequences in favour of those matching the reference to which they were mapped. In sample C1, the relative frequency of the most abundant haplotype, here referred to as haplotype 1, increased by 7.8 percentage points between sequences mapped to the least similar reference sequence compared to sequences mapped to a reference identical to the haplotype (Figure 3). For C2, the difference was 6.0%. The most abundant compatible haplotype pair (consisting of four variant sites for C1 and three variant sites for C2) made up 74.6–79.6% and 79.3–81.1% of the reads between the alignments to different reference sequences. Thus, for both samples, the two most frequent haplotypes remained the most frequent haplotypes irrespective of the reference sequence used in the alignment.

Alternative mRNA splicing accompanies two of the four commonly studied *DPYD* variants (c.1905+1G>A and c.1129-5923C>G). To determine if alternative splicing affects the mapping of results and genotype calls when RNA is used as an initial template for genotyping with long-range sequencing, we performed additional analyses in carriers of these variants. Both C1 and C2, as well as a third sample, C14, were heterozygous for the deep intronic variant (c.1129-5923C>G), which leads to alternatively spliced copies with a 44 bp insert in exon 11. Therefore, the sequences were additionally mapped to a reference sequence including the 44 bp insert. In sample C1, 4.1% (36/876) of the reads containing the c.1129-5923C>G variant also included a 44 bp insertion, while it was 3.0% (40/1353) of the reads in C2, and 2.2% (28/1255) in C14. The absolute numbers remained unchanged when mapping the sequences to an alternative reference sequence, which included the 44 bp insert. In addition, one patient sample was a carrier of the c.1905+1G>A *DPYD* risk-variant leading to the deletion of exon 14, corresponding to 165 bp. Of the 1371 aligned reads, 58.0% (795/1371) contained the deletion when aligned to hg38, while 57.9% (794/1371) contained the deletion when aligned to an alternative reference lacking the exon 14 sequence. These results are consistent with the previous finding that c.1129-5923C>G and c.1905+1G>A result in non-obligate and obligate alternative splicing, respectively [20].

### 2.5. Haplotype Phasing

Our previous studies identified that haplotype structure between multiple *DPYD* variants could modulate the impact of coding region variants [14] and established that *cis*- or *trans*-conformation of variants could affect variant impacts on DPD enzyme activity [21]. These findings indicate that haplotype phasing is an important consideration for *DPYD*, similarly to other pharmacogenes such as *TPMT* [22]. Therefore, we sought to assess the suitability of our Nanopore-based long-range sequencing strategy for haplotype phasing across the coding region of *DPYD*.

**Figure 4:**
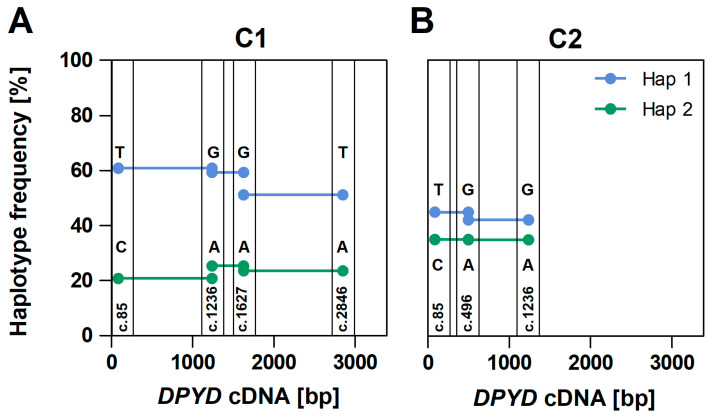
Nearest neighbour tiling approach applied to phase haplotypes in samples C1 (**A**) and C2 (**B**). The frequency of the combination of bases occurring together on a read in neighbouring variant loci is used to determine each two-locus haplotype. Variants plotted according to their positions in 3384 bp *DPYD* transcript amplicons. Full-length haplotypes are then constructed by joining two-loci haplotypes if they overlap with an identical basecall, indicated with corresponding colours in the figure.

The following approach to determine the full-length cDNA *DPYD* haplotypes was applied. In cases with more than two variants in a sample, all compatible two-locus haplotype pairs were determined based on their relative frequencies. As can be seen in Figure 2A, the frequency of PCR chimeras increases with the distance between two variant positions. Thus, we used a nearest neighbour tiling approach as depicted in Figure 4. In more detail, in case of three variant positions, the two-locus haplotypes involving the SNP located in the middle and the two outer SNPs are determined by accepting the compatible haplotype pair occurring at the highest frequency. Then the two-locus haplotype pair is joined based on the matching overlapping base. With more than three SNPs present, the procedure is repeated, starting from one end of the amplicon, then stepwise joining the next two-locus-haplotype with the 2 + n-locus haplotype based on their matching overlapping base. A failure of obtaining matching overlapping bases at any step of the procedure is considered as being indicative of excessive PCR chimera formation, which is potentially biasing haplotype phasing. The maximum number of variants found in one of the 21 individuals was four. A full overview of all variants including phasing results can be found in Supplementary Table S1. Using this procedure, we could determine the full-length-cDNA haplotypes of all samples with our standard PCR protocol consisting of 35 amplification cycles and 8 ng/µL template cDNA input. Based on this PCR protocol, we observed a compatible two-locus PCR chimera pair of neighbouring SNPs at a frequency of 3.9–34.8%, while the true haplotypes made up 61.9–91.7% of the reads. In samples extracted from blood, the true haplotypes made up 75.4–91.7% of the reads, while the PCR chimeric haplotypes ranged between 3.9 and 17.3%.

### 2.6. Haplotype Phasing Applied to Two Clinical Cases

The first case involves an 80-year-old male who was diagnosed with stage IIIB colorectal cancer. The patient was treated with adjuvant chemotherapy with capecitabine monotherapy, intended to last 6 months. The patient developed severe oral and gastrointestinal mucositis (Common Terminology Criteria for Adverse Events [CTCAE] Grade 3), as well as severe prolonged neutropenia (CTCAE Grade 4, lowest absolute blood neutrophil count [ANC] was at 0.03 × 10^9^ G/L) shortly thereafter, and the treatment was put on hold at day 8 of therapy. Symptoms did not improve after therapeutic discontinuation, and he was hospitalised at day 13. The patient remained hospitalised for the following 3 weeks, at which time symptoms had resolved, and he was discharged. The patient was retrospectively genotyped for *DPYD* c.1905+1G>A, c.1679T>G, c.2846A>T, and c.1129-5923C>G and found to be heterozygous for variant alleles at c.1129-5923 and c.2846.

The second case involves a 61-year-old male who presented with intermediate-stage adenocarcinoma of the rectum (mrT3ab, mrN0, CRM negative, EMVI negative). Immunohistochemistry revealed that the tumour was microsatellite stable (MSS) and carried a mutation in *KRAS*. Preoperative long-course chemoradiotherapy was recommended by the tumour board since the tumour was located 6 cm from anal verge measured by rigid endoscopy. The patient was prospectively genotyped for the four *DPYD* risk variants tested in patient 1, and was found to be heterozygous for the two risk variants c.1679T>G and c.1905+1G>A. 

According to the Swiss Group of Pharmacogenomics and Personalised Therapy (SPT), the recommendations for the first patient would be to start at 25% of the initially recommended dose, administered through infusion followed by therapeutic drug monitoring, while the second patient would not be advised to receive any FP-based therapy. These recommendations are based on the assumption that the variants are in *trans*. In the event of the variants being in *cis*, both patients could be treated as carriers of a single non-functional allele, with a recommended 50% reduction in starting dose. We therefore haplotype-phased the two patients using the Nanopore-based full length *DPYD* amplicon sequencing method. The variants were found to be in *trans* configuration in both patients (Table 1).

After initial treatment, the first patient developed three resectable metachronous liver metastases. Based on the two decreased-function variants identified following initial FP therapy, he was started on a treatment with 25% of the recommended starting dose according to body surface area of 5-fluorouracil and a full dose of Oxaliplatin (FOLFOX), combined with therapeutic drug monitoring (TDM). First AUC measurement was 3.6 mg h/L (recommended 20–30 mg h/L). The starting dose was increased by 15% per cycle for the subsequent two cycles, resulting in AUC values of 18 mg h/L and 16.8 mg h/L, respectively. Although AUC had not reached the target range, the dose of 5-fluorouracil was maintained at this level (33% of full dose) due to increased toxicity. The patient responded well to the treatment, and hepatic metastasectomy was performed.

Based on the genotype of the second patient, the planned concomitant capecitabine could not be given. The patient was treated with short course 5 × 5 Gy radiation with a prolonged surgery wait of 11 weeks. The tumour could be resected R0, and the pathological was ypT3ypN0 (0/17), L0, V0, pN0. At 19 months after surgery for the primary tumour, the patient underwent wedge resection of a suspicious pulmonary nodule. A metachronous metastasis of the rectal adenocarcinoma was histologically confirmed.

## 3. Discussion

We successfully developed a genotyping method based on full-length cDNA amplicon Nanopore sequencing for fully phased resequencing of the entire coding region of the *DPYD* pharmacogene. With our method, we could confirm all *a priori* detected 37 heterozygous and 104 homozygous wt genotypes in 21 individuals and confirmed *a posteriori* 11 additional heterozygous genotypes detected with Nanopore sequencing. Furthermore, we obtained fully phased genotypes for all individuals, including two clinical cases. Targeted long-read amplicon sequencing has shown potential as a tool for generating phased sequence-based genotype data for disease-associated genes and pharmacogenes [23,24,25,26]. However, long-read amplicon sequencing is vulnerable to considerable technical bias, including the formation of PCR chimeras and alignment bias [27]. We showed that in the case of full-length cDNA sequencing of *DPYD*, PCR chimeras are generated at a considerable rate even under our most stringent PCR conditions. This high degree of PCR chimerism may be critical for applying phasing algorithms that have been originally developed for shotgun next-generation sequencing (NGS) data [28,29]. Indeed, when using the software *WhatsHap* v2.1 as an example for phasing, we did not always observe concordant results with different PCR conditions for the same individuals. Since the coding region of the targeted gene *DPYD* in general only contains small differences compared to the reference, here we observed a maximum of four polymorphisms in the 3384 bp-long amplified coding region per individual in a given sample; we did not observe any important alignment bias with regard to the inferred haplotypes. We did not measure any bias when using samples carrying the deep intronic *DPYD* risk variant c.1129-5923C>G, when we aligned them to either the reference genome or an alternative reference including the 44 bp insertion. Neither did we experience any remarkable changes when aligning a sample lacking exon 14 to a reference sequence that excluded the 165 bp corresponding to exon 14. These locus-specific features make *DPYD* amenable to a very simplified genotyping and phasing bioinformatics pipeline as reported here.

To filter out incomplete PCR amplicons that did not span across the entire gene and therefore all variants of interest, we excluded sequences shorter than 3100 bp (of 3384 bp). Although the filtering came at the risk of removing reads with large deletions (>284 bp), all *DPYD* exons were between 69–216 bp; therefore, all reads containing a single exon-skipping event should be retained. Upon sequencing an individual heterozygous for the c.1905+1G>A *DPYD* risk variant, a deletion of 165 bp corresponding to exon 14 was directly observable in 58.0% of the reads upon visual inspection using IGV. 

It is noted that adjusting the length filtering could permit the detection of even larger deletions; however, allelic dropouts—e.g., due to mismatches in the primer regions—could not be detected with this approach. Since our method is based on mRNA, mutations that introduce premature termination codons and the transcripts of the affected strand may, consequently, become subject to nonsense-mediated mRNA decay (NMD). This situation can theoretically result in an allelic drop-out at the mRNA level. In the present study, all individuals were heterozygous for one or more variants; we therefore concluded that both alleles were present in all individuals. 

Interestingly, this finding also applies to the included carriers of the most common risk variant, the deep intronic c.1129-5923C>G mutation, which acts as a cryptic splice donor site, resulting in a 44 bp insertion in exon 11 leading to a reading frameshift and a premature termination codon in exon 11 [30]. All carriers of this mutation could be identified by the presence of the synonymous mutation in the coding region c.1236G>A, which is in linkage disequilibrium with c.1129-5923C>G and is frequently used as a surrogate marker for c.1129-5923C>G in clinical practice. However, a recent study reported cases of rare recombinants of the two variants, demonstrating that c.1236G>A is not in perfect linkage disequilibrium and can thus, on rare occasions, occur without c.1129-5923C>G on the same strand [31]. This could be accounted for within our approach by using the following modification of our protocol: to confirm the presence of c.1129-5923C>G by a targeted method once the c.1236G>A variant is detected, or to add a PCR amplicon of the intronic region directly in our assay. Interestingly, we were able to directly observe the alternatively spliced cDNA containing 44 bp insertion in all three carriers of c.1129-5923C>G, although at very low frequencies (2.2–4.1%) of the aligned reads, which suggests that NMD does not completely eliminate the mis-spliced mRNAs. Whether this represents a consistent pattern for c.1129-5923C>G carriers in general and could be reliably used to confirm the presence of the causal c.1129-5923C>G variant needs further investigation.

An advantage of our method over conventional genotyping approaches is that it allows for genotype phasing of presumably compound heterozygous cases, as illustrated by the two patient cases. Although patients carrying more than one *DPYD* risk variant are relatively rare, they have frequently been reported in the literature, especially in the context of severely increased risk of toxicity and lethal adverse reactions [31,32,33,34,35]. Conventional sequencing methods may struggle to resolve the phase of the variants accurately in such cases. Long-read sequencing overcomes this limitation by providing reads spanning the entire region, allowing for the unambiguous phasing of multiple variants. This is crucial for interpretation in cases where the interactions between the variants contribute to the clinical outcome. While the use of fluoropyrimidines is not recommended for patients with two non-functional alleles in a *trans* configuration, those carrying the two variants in a *cis* configuration could tolerate a lower dosage.

The ability to generate completely phased *DPYD* genotypes is also of great relevance with regard to current research focusing on the relevance of more common coding *DPYD* variants and *cis*-regulatory polymorphisms with regard to the inter-individual response to FP treatment. For example, a recent study showed that in endogenous product substrate ratio in carriers of common polymorphisms in *DPYD*, c.85T>C (rs1801265, MAF = 0.227) and c.496A>G (rs2297595, MAF = 0.110) and the deep intronic risk variant c.1129-5923C>G (rs75017182, MAF = 0.024) were dependent on haplotype structure [14]. In this study, endogenous plasma dihydrouracil:uracil (UH_2_:U; product: substrate) ratios at the population level were used as a surrogate marker for systemic DPD function in healthy volunteers. Different haplotypes were associated with different mean UH_2_:U ratios, potentially explaining the conflicting results that have been reported for these variants in the context of FP toxicity risk [14]. A subsequent study retrospectively analysing a Canadian cancer cohort reported a similar finding for associations with FP-related toxicity, although some differences concerning particular haplotypes were noted [36]. In both studies, the haplotypes were inferred statistically based on linkage disequilibrium patterns. In these cases, our method can provide a more accurate means to determine the true haplotype composition in these individuals, enhancing the accuracy of future studies on this topic. 

From a diagnostic perspective, we suggest that the main application of our method is genotype phasing presumably compound heterozygous cases. However, the method presented here may also be suitable for genotyping the complete coding region of *DPYD* in routine diagnostics, given the sample throughput is sufficiently high to allow for an improved turnaround time and cost effectiveness. An internal assessment revealed that if a sufficient number (≥12) of individuals are analysed in a single Nanopore sequencing run, the presented method outperformed our routine method based on Sanger sequencing of all exons with regard to hands-on time, costs, and turnaround time. Of course, since there are different cost structures in different countries, this assessment may not apply to other situations. Our method requires up to three days for RNA extraction, library preparation, sequencing, and subsequent analysis, which is compatible with the recommended turnaround time of up to 7 days in order to avoid therapy delays (period from blood draw to results reported) [37,38,39,40]. Also, the increase in sensitivity may vary from laboratory to laboratory depending on the patient population. For example, based on allele frequencies in the gnomADatabase [41], ca. 0.08% of the European population would be carriers of an additional 28 variants in *DPYD* that have been classified to confer decreased or no enzyme function by CPIC [42]. In contrast, the increase in sensitivity would be considerably higher in other populations [10]. 

Since the assay is based on full-length cDNA amplicons, we are able to obtain single reads that span the entire coding region of *DPYD* with a single PCR reaction. However, with regard to routine diagnostics, our method is pre-analytically more challenging than tests based on genomic DNA from whole blood samples, since collection tubes containing RNA-stabilising agents are required to prevent the degradation of the much-less-stable RNA. In this context, it is worth mentioning that our data set includes a small preliminary experiment involving one individual, for whom we successfully sequenced full-length *DPYD* based on RNA extracted from buffy-coat from blood samples collected in EDTA tubes. Buffy-coat was isolated from whole blood either immediately after collection and stored at −80 °C, or the whole blood samples were left at ambient temperature for up to 72 h, after which buffy-coat was isolated. RNA was subsequently extracted from the buffy-coats (Table S4). We furthermore successfully sequenced two additional individuals using RNA isolated from frozen buffy-coat that had been stored at −80 °C up to 8 months (Table S1). 

With the current protocol, we reliably called variants and haplotype-phased samples with as few as 619 high-quality full-length amplicon reads. However, the lower limits for haplotype phasing have not been established. Interestingly, the existing literature suggests that reliable results can be achieved with as few as 60 reads, underscoring the potential of our method [43]. With an average of 44,796 sequencing reads per sample, we see ample room for further technical improvement. Future optimisation steps could involve increasing stringency in read filtering to enhance the quality further, increasing the quality of the input, and reducing PCR chimerism by refining the current PCR protocol. Moreover, there is potential for cost-effective scaling through multiplexing and parallel sequencing of multiple genes, given the necessary read depth is retained, as was done in Liau et al. [44].

## 4. Materials and Methods

### 4.1. Liver Tissue and Blood Samples

RNA samples from liver tissue and blood samples were included, from which *DPYD* genotyping data were available and for which the additional analyses were in the scope of the ethical approvals. Liver tissue samples consisted of leftover material obtained by the University Clinic of Visceral Surgery and Medicine from patients who consented to the use of their specimens for general research with a signed general consent at Inselspital (University Hospital of Bern, Bern, Switzerland, KEK-BE: 2016-02202). Anonymized blood samples for method development were obtained from healthy blood donors (University Hospital of Bern, Bern, Switzerland, KEK-BE: Req-2020-00173). In addition, blood samples from two patients were included. Informed patient consent was obtained by their treating physicians.

### 4.2. DNA Extraction

DNA was extracted from 2 mL of whole blood collected in EDTA tubes using the QIAamp DNA Blood Mini Kit (QIAGEN, Hilden, Germany) following the manufacturer’s protocol for blood extraction. The DNA was eluted in 400 µL elution buffer. Liver tissues (~5 µg) were homogenised in 80 µL phosphate-buffered saline (PBS, pH 7.4) using 3 mm steel beads at <25 Hz for ~3 min using a CryoMill TissueLyser (Retsch^®^, Frankfurt, Germany), and DNA was subsequently extracted using the QIAamp Blood Mini Kit (QIAGEN, Hilden, Germany) following the manufacturer’s protocol for tissue extraction. DNA was quantified using a Nanodrop™ One spectrophotometer (Thermo Fisher Scientific, Waltham, MA, USA).

### 4.3. RNA Extraction

RNA was extracted from tubes containing RNA-stabilised whole blood, as well as from fresh and frozen buffy-coat from whole blood collected in EDTA tubes, and from liver tissue. PAXgene Blood RNA tubes and kit (QIAGEN, Hilden, Germany) were used to collect and extract RNA-stabilised blood samples, as per manufacturer’s instructions. For a full overview, refer to Table S1. Extracted PAXgene Blood RNA was eluted with 40 µL Buffer BR5, twice (final volume 80 µL). RNA was furthermore extracted from 500 µL buffy-coat derived from 7 mL whole blood collected in EDTA tubes. The whole-blood samples were either immediately centrifuged at 2000× *g* for 15 min at 4 °C, after which the buffy-coat was collected and cryopreserved at −80 °C for up to 8 months, or the EDTA samples were left at ambient temperature for 0–72 h, after which the buffy-coat was collected. RNA was then isolated from buffy-coat using QIAzol (QIAGEN, Hilden, Germany) lysis buffer containing phenol added to the buffy-coat in a 1:1 ratio, after which one part chloroform (99.0–99.4% purity, Merck, Darmstadt, Germany) was added to every five parts QIAzol for phenol-chloroform separation. Hereafter, RNA was extracted from the upper aqueous phase using the RNeasy Kit (QIAGEN, Hilden, Germany), following the manufacturer’s protocol. The RNA was eluted twice with the same 30 µL RNase-free H_2_O (final volume 30 µL). RNA from liver was extracted from ~5 µg of tissue. The tissue was homogenised in 700 µL QIAzol lysis reagent (QIAGEN, Hilden, Germany) using 3 mm steel beads in the CryoMill TissueLyser (Retsch^®^, Frankfurt, Germany) at <25 Hz for ~3 min. The lysate was incubated for 5 min at ambient temperature, after which 140 µL chloroform (Merck, Darmstadt, Germany) was added, and RNA was isolated from the upper aqueous phase utilizing the RNeasy Kit (QIAGEN, Hilden, Germany) in accordance with the manufacturer’s instructions. Lastly, the RNA from liver was eluted in 100 µL H_2_O, followed by an additional 100 µL H_2_O elution step (final volume 200 µL). RNA concentrations were measured using the NanoDrop™ One spectrophotometer (Thermo Fisher Scientific, Waltham, MA, USA), and samples were stored at −80 °C until used.

### 4.4. cDNA Synthesis

First-strand cDNA was synthesised from 1 mg of RNA template with SuperScript™ IV Reverse Transcriptase (Thermo Fisher Scientific, Waltham, MA, USA) using Oligo(dT)_20_ primers (Invitrogen, Waltham, MA, USA), including an RNAse inhibition step with RNaseOUT™ Recombinant Ribonuclease Inhibitor (Thermo Fisher Scientific, Waltham, MA, USA) in a reaction volume of 20 µL. The cDNA synthesis was performed according to the manufacturer’s protocol, with the following specific modifications: incubation for 10 min at 50 °C in a Biometra Trio thermal cycler (Labgene Scientific, Châtel-Saint-Denis, Switzerland) before inactivating the enzyme for 10 min at 80 °C. The efficacy of the conversion was confirmed by agarose gel electrophoresis (0.8%) using 5 µL of the cDNA synthesis reaction mixture, while the residual 15 µL was purified using the QIAquick PCR Purification Kit (QIAGEN, Hilden, Germany) and eluted in 30 µL elution buffer after a 5 min on-column incubation. In the next step, the eluate was re-added to the same column, followed by an additional 5 min incubation before re-elution (final volume 30 µL).

### 4.5. Long-Range PCR Amplification of DPYD cDNA 

The full-length *DPYD* transcript was amplified in a total PCR reaction volume of 25 µL containing JumpStart™ REDAccuTaq^®^ LA DNA-Polymerase (Sigma Aldrich, St. Louis, MO, USA), 0.4 µM each of *DPYD* specific primers (forward 5′-CGCAAGGAGGGTTTGTCACTG-3′, reverse 5′-GAACATCCAATTAACTGCCACAC-3′; Supplementary Table S5; Microsynth AG, Balgach, Switzerland), 0.4 mM dNTP mix (Promega, Madison, WI, USA), and 4 µL cleaned cDNA eluate as template. The expected product size was 3384 bp, corresponding to a region 47 nucleotides upstream of the start codon through 259 nucleotides downstream of the stop codon, in the untranslated regions of the mRNA. PCR conditions included an initial denaturation step of 3 min at 95 °C followed by 35 amplification cycles consisting of a denaturation step of 15 sec at 94 °C, annealing step for 15 sec at 55 °C, and extension for 4 min at 68 °C. A final extension step of 6 min at 68 °C was used. Modifications to these cycling parameters were evaluated, which included adjustment to the number of amplification cycles (30, 35, and 40) and varying amounts of input template (4, 8, and 16 ng/µL), as described in conjunction with relevant results. Amplicon size was confirmed by agarose gel electrophoresis (0.8%) prior to purification using a QIAquick PCR Purification Kit (QIAGEN, Hilden, Germany). Purified amplicons were eluted in 30 µL elution buffer EB after a 5 min incubation on column. The eluate was re-added to the column and incubated for 5 additional min before collection.

### 4.6. Targeted Genotyping

Genotyping had been carried out in the context of previous studies and routine diagnostics, and as such, not all variants were targeted in all individuals. It was carried out on the QuantStudio 6 Flex Real-Time PCR System (Applied Biosystems, Waltham, MA, USA) using DNA as template, TaqMan SNP Genotyping Assays, and TaqMan 2× Universal PCR Master Mix (Thermo Fisher Scientific, Waltham, MA, USA). Assays for the following *DPYD* variants were used (SNP ID, assay ID): c.85T>C (rs1801265, C___9491497_10), c.496A>G (rs2297595, C__16187014_20), c.1129-5923C>G (rs56293913, C__25596097_10), c.1601G>A (rs1801158, C___8383855_20), c.1627A>G (rs1801159, C___1823316_20), c.1679T>G (rs55886062, C__11985548_10), c.1905+1G>A (rs3918290, C__30633851_20), c.2194G>A (rs1801160, C__11372171_10), c.2846A>T (rs67376798, C__27530948_10).

### 4.7. Long-Read DPYD Amplicon Sequencing 

Sequencing libraries were prepared from PCR products using the Ligation Sequencing Kit SQK-LSK109 (Oxford Nanopore Technologies, Oxford, UK) and were natively barcoded with NBD104 and NBD114. Barcoded samples were sequenced using a total of four MinION flow cells (FLO-MIN106, R9.4.1 chemistry; Oxford Nanopore Technologies) at the IFIK NGS platform of the Institute for Infectious Diseases (IFIK, Inselspital, University Hospital of Bern, University of Bern, Bern, Switzerland) using a GridION X5 instrument (Oxford Nanopore Technologies, Oxford, UK). The basecalling process was performed in real time using ont-guppy-for-gridion (v. 3.2.10-1; high accuracy mode) (Oxford Nanopore Technologies, Oxford, UK) at a speed of 450 bases per second (bps) while simultaneously filtering the sequence reads and retaining only those with a mean Guppy Q-score of ≥9. On average, 44,796 (range 2757–91,848) sequence reads were generated per sample; passing reads had a mean Q-score of 12.8. Sequence read statistics were calculated using *NanoPlot* (v. 1.42.0) and summarised with *MultiQC* (v. 1.21).

### 4.8. Sequencing Read Processing and Genotyping

A subset of 4000 sequence reads per sample was used for analysis, except for one where only 2757 sequence reads were obtained (all reads were used). Adapters and barcodes were removed with *Porechop* (v. 0.2.4), and sequences upstream of the 5′-ends of primers were trimmed with *Cutadapt* (v. 4.5). Only reads that included both primers and, therefore, were expected to span across the full target region were of interest, while the rest of the reads were discarded. Reads that were shorter than 3100 bp were also filtered out, still allowing for potential sequencing errors, inserts, and some splicing variants to be considered (range 619–3320 reads per sample, mean = 2315, median = 2433). *Minimap2* (v. 2.26) with the ‘*-ax map-ont*’ flag was used to align the processed reads to reference sequence NM_000110.4 (Homo Sapiens *DPYD*, transcript variant 1, mRNA, GRCh38/hg38) aligning 100% of the reads with the reference. The aligned reads were filtered, sorted, and indexed using *samtools* (v. 1.15.1, [45]) with the ‘*view --no-PG –hT*’ setting. Variant positions were determined from pileup data using a predefined threshold of 20% variant allele frequency with respect to the reference sequence and visually inspected using the Interactive Genomic Viewer (IGV) (v. 2.15.2) [46]. A flowchart with an overview of the sample preparation, sequencing, and bioinformatics pipeline can be found in Supplementary Materials Figure S1. 

All additional variants detected with Nanopore were confirmed by targeted TaqMan genotyping using DNA or Sanger sequencing carried out on a 3130xl Genetic Analyzer (Applied Biosystems, Waltham, MA, USA) using cDNA as template. The protocol used was previously described in [47]. The sequences of the primers can be found in Supplementary Table S5. 

### 4.9. Identification of PCR Chimeras

Basecalls were extracted from all heterozygous variant positions within aligned BAM files using the *Rsamtools* (v. 2.16.0) and *GenomicAlignment* packages (v. 1.36.0) via *BiocManager* (v.1.30.21.1) in RStudio (2023.06.0+421), R (v. 4.3.1, 2023-06-16 ucrt). The relative frequency of all two-locus variant combinations of the aligned reads of a sample were calculated, i.e., the fraction of reads in which each base at a variant position occurred in combination with another base at a second variant position. The four possible haplotypes for each variant pair were sorted according to their frequency, with haplotype 1 being the most frequent haplotype. The two-locus haplotypes were subsequently grouped in pairs according to their compatibility. The true two-locus haplotype pairs were identified as they occurred at a relative frequency of >60%; the alternative pairs were consequently regarded as PCR chimeras based on the following assumption: with increased rates of chimerism, the early-generation chimeric haplotypes will recombine back to the original state at an increasing rate, preventing certain haplotypes from becoming dominant in frequency. Thus, in the worst case, we expected to observe both two-locus haplotype pairs at equal frequencies. In this situation, one cannot differentiate between the true and chimeric haplotypes.

### 4.10. Statistics

All figures and trend lines were generated using GraphPad Prism 5 (v.5.02) and R (v. 4.3.1, 2023-06-16 ucrt) using RStudio (2023.06.0+421). A multivariable logistic regression model was employed to analyse the effects of PCR amplification cycles and input conditions on PCR recombination. The analysis was conducted using the glm function from the stats package in R (v. 4.3.1, 2023-06-16 ucrt).

## 5. Conclusions

We developed a time- and cost-effective method suitable for genotyping the complete coding region and providing completely phased genotypes of the important pharmacogene *DPYD*. Genotype phasing is of particular importance for carriers of more than one risk variant, where a *cis*- or a *trans*-configuration of the risk variants would translate into different toxicity risks and dosing recommendations. As recent studies indicated that haplotype structure may influence DPD function and consequently the individual FP-related toxicity risk in cancer patients, fully phased genotypes will also be of great importance for future research on this topic.

## Figures and Tables

**Table 1:** Haplotype-phased variants in *DPYD* in cancer patients with multiple variants. *DPYD* risk variants are in **bold**. Variant alleles are indicated in **bold,** underlined capital letters.

*DPYD* Variant	Patient 1		Patient 2	
	Strand 1	Strand 2	Strand 1	Strand 2
c.85	T	**C**	t	t
**c.1129-5923**/c.1236 *	A	**G**	a	a
c.1627	**G**	A	a	a
**c.1679**	t	t	**G**	T
**c.1905+1**	g	g	G	**A**
**c.2846**	**T**	A	a	a
Recommendation	25% of starting dose		No FP therapy	

* The c.1236A>G is used as a surrogate marker for the c.1129-5923C>G.

## Data Availability

Data are contained within this article or Supplementary Materials.

## Supplementary Materials

The following supporting information can be downloaded at: https://www.mdpi.com/article/10.3390/ijms25147599/s1.
