# Supplementary material for: Approach for Phased Sequence-Based Genotyping of the Critical Pharmacogene Dihydropyrimidine Dehydrogenase (DPYD)
Source: Int J Mol Sci. 2024 Jul 11;25(14):7599. doi: 10.3390/ijms25147599 (PMC11277299; doi:10.3390/ijms25147599)
Supplement: Supplementary file 1 [file ijms-25-07599-s001.zip › ijms-3060383-Supplementary materials.pdf]

Supplementary materials

Approach for Phased Sequence-Based Genotyping of the Critical Pharmacogene Dihydropyrimidine Dehydrogenase (*DPYD*)

Alisa Ambrodji [1,2], Angélique Sadlon [1], Ursula Amstutz [1], Dennis Hoch [3], Martin D. Berger [3], Sara Bastian [4], Steven M. Offer [5] and Carlo R. Largiadèr [1,*]

[1] Department of Clinical Chemistry, Inselspital, University Hospital of Bern, University of Bern, INO-F, 3010 Bern, Switzerland
[2] Graduate School for Cellular and Biomedical Sciences, University of Bern, 3012 Bern, Switzerland
[3] Department of Medical Oncology, Inselspital, University Hospital of Bern, 3010 Bern, Switzerland
[4] Department of Medical Oncology, Cantonal Hospital Graubünden, 7000 Chur, Switzerland
[5] Department of Pathology, Carver College of Medicine, University of Iowa, Iowa City, IA 52242, USA
* Correspondence: carlo.largiader@insel.ch

Supplementary Materials:

Table S1. Phased *DPYD* haplotypes in 21 subjects sequenced with Oxford Nanopore Technologies. 0 = reference allele, 1 = alternative allele. Heterozygous sites indicated in bold. Phasing represented by pipe (vertical bar) ∣ symbol. All variants on the left side of the pipe belong to one haplotype, while all on the right side to the second haplotype. Asterisk (*) symbol indicates 11 *a posteriori* confirmed heterozygous sites which were initially identified with full length sequencing of the *DPYD* transcript.

<table>
<tr><th></th><th></th><th colspan="21">Subject Nº</th></tr>
<tr><th></th><th></th><th>C1</th><th>C2</th><th>C3</th><th>C4</th><th>C5</th><th>C6</th><th>C7</th><th>C8</th><th>C9</th><th>C10</th><th>C11</th><th>C12</th><th>C13</th><th>C14</th><th>C15</th><th>C16</th><th>C17</th><th>C18</th><th>C19</th><th>C20</th><th>C21</th></tr>
<tr><td rowspan="11">DPYD variant</td><td>c.85T>C</td><td>1 | 0</td><td>1 | 0</td><td>0 | 0</td><td>1 | 0</td><td>1 | 0</td><td>1 | 0</td><td>0 | 0</td><td>1 | 0</td><td>0 | 0</td><td>1 | 0</td><td>0 | 0</td><td>1 | 0</td><td>1 | 0</td><td>1 | 0</td><td>1 | 0</td><td>1 | 0</td><td>1 | 0</td><td>1 | 0</td><td>1 | 0</td><td>0 | 0</td><td>1 | 0*</td></tr>
<tr><td>c.496A>G</td><td>0 | 0</td><td>0 | 1</td><td>0 | 0</td><td>0 | 0</td><td>1 | 0</td><td>1 | 0</td><td>0 | 0</td><td>1 | 0</td><td>0 | 0</td><td>0 | 0</td><td>0 | 0</td><td>1 | 0</td><td>1 | 0</td><td>0 | 0</td><td>1 | 0</td><td>1 | 0</td><td>0 | 0</td><td>1 | 0</td><td>1 | 0</td><td>0 | 0</td><td>0 | 0</td></tr>
<tr><td>c.775A>G</td><td>0 | 0</td><td>0 | 0</td><td>0 | 0</td><td>1 | 0*</td><td>0 | 0</td><td>0 | 0</td><td>0 | 0</td><td>0 | 0</td><td>0 | 0</td><td>0 | 0</td><td>0 | 0</td><td>0 | 0</td><td>0 | 0</td><td>0 | 0</td><td>0 | 0</td><td>0 | 0</td><td>0 | 0</td><td>0 | 0</td><td>0 | 0</td><td>0 | 0</td><td>0 | 1*</td></tr>
<tr><td>c.1236G>A</td><td>1 | 0</td><td>1 | 0</td><td>0 | 0</td><td>0 | 0</td><td>0 | 0</td><td>0 | 0</td><td>0 | 0</td><td>0 | 0</td><td>0 | 0</td><td>0 | 0</td><td>0 | 0</td><td>0 | 0</td><td>0 | 0</td><td>1 | 0</td><td>0 | 0</td><td>0 | 0</td><td>0 | 0</td><td>0 | 0</td><td>0 | 0</td><td>0 | 0</td><td>0 | 0</td></tr>
<tr><td>c.1601G>A</td><td>0 | 0</td><td>0 | 0</td><td>0 | 0</td><td>0 | 0</td><td>0 | 0</td><td>0 | 0</td><td>0 | 0</td><td>0 | 0</td><td>0 | 0</td><td>0 | 0</td><td>0 | 0</td><td>0 | 0</td><td>0 | 0</td><td>0 | 0</td><td>0 | 0</td><td>0 | 0</td><td>0 | 0</td><td>0 | 0</td><td>0 | 0</td><td>0 | 0</td><td>0 | 0</td></tr>
<tr><td>c.1627A>G</td><td>0 | 1*</td><td>0 | 0</td><td>0 | 0</td><td>0 | 0</td><td>0 | 0</td><td>0 | 1</td><td>0 | 0</td><td>0 | 0</td><td>1 | 0</td><td>0 | 1</td><td>0 | 0</td><td>0 | 0</td><td>0 | 0</td><td>0 | 0</td><td>0 | 0</td><td>0 | 0</td><td>0 | 0</td><td>0 | 0</td><td>0 | 1*</td><td>1 | 0*</td><td>1 | 0*</td></tr>
<tr><td>c.1679T>G</td><td>0 | 0</td><td>0 | 0</td><td>1 | 0</td><td>0 | 1</td><td>0 | 0</td><td>0 | 0</td><td>0 | 0</td><td>0 | 0</td><td>0 | 0</td><td>0 | 0</td><td>0 | 0</td><td>0 | 0</td><td>0 | 0</td><td>0 | 0</td><td>0 | 0</td><td>0 | 0</td><td>0 | 0</td><td>0 | 0</td><td>0 | 0</td><td>0 | 0</td><td>0 | 0</td></tr>
<tr><td>c.1896T>C</td><td>0 | 0</td><td>0 | 0</td><td>0 | 0</td><td>0 | 0</td><td>0 | 0</td><td>0 | 0</td><td>1 | 0*</td><td>0 | 0</td><td>0 | 0</td><td>0 | 0</td><td>1 | 0*</td><td>0 | 0</td><td>0 | 1*</td><td>0 | 0</td><td>0 | 0</td><td>0 | 0</td><td>0 | 0</td><td>0 | 0</td><td>0 | 0</td><td>0 | 0</td><td>0 | 0</td></tr>
<tr><td>c.1905+1G>A</td><td>0 | 0</td><td>0 | 0</td><td>0 | 1</td><td>0 | 0</td><td>0 | 0</td><td>0 | 0</td><td>0 | 0</td><td>0 | 0</td><td>0 | 0</td><td>0 | 0</td><td>0 | 0</td><td>0 | 0</td><td>0 | 0</td><td>0 | 0</td><td>0 | 0</td><td>0 | 0</td><td>0 | 0</td><td>0 | 0</td><td>0 | 0</td><td>0 | 0</td><td>0 | 0</td></tr>
<tr><td>c.2194G>A</td><td>0 | 0</td><td>0 | 0</td><td>0 | 0</td><td>0 | 0</td><td>0 | 0</td><td>0 | 0</td><td>0 | 0</td><td>0 | 0</td><td>0 | 0</td><td>0 | 0</td><td>0 | 1</td><td>0 | 0</td><td>0 | 0</td><td>0 | 0</td><td>0 | 0</td><td>1 | 0*</td><td>0 | 0</td><td>0 | 0</td><td>0 | 0</td><td>0 | 0</td><td>0 | 0</td></tr>
<tr><td>c.2846A>T</td><td>0 | 1</td><td>0 | 0</td><td>0 | 0</td><td>0 | 0</td><td>0 | 0</td><td>0 | 0</td><td>0 | 0</td><td>0 | 0</td><td>0 | 0</td><td>1 | 0</td><td>0 | 0</td><td>0 | 0</td><td>0 | 0</td><td>0 | 0</td><td>0 | 0</td><td>0 | 0</td><td>0 | 0</td><td>0 | 0</td><td>0 | 0</td><td>0 | 0</td><td>0 | 0</td></tr>
<tr><td rowspan="4">RNA source</td><td>Liver</td><td></td><td>x</td><td></td><td>x</td><td>x</td><td>x</td><td>x</td><td>x</td><td>x</td><td>x</td><td>x</td><td>x</td><td>x</td><td>x</td><td>x</td><td>x</td><td></td><td></td><td></td><td></td><td></td></tr>
<tr><td>PAXgene</td><td>x</td><td></td><td>x</td><td></td><td></td><td></td><td></td><td></td><td></td><td></td><td></td><td></td><td></td><td></td><td></td><td></td><td>x</td><td></td><td></td><td></td><td></td></tr>
<tr><td>Fresh buffy-coat</td><td></td><td></td><td></td><td></td><td></td><td></td><td></td><td></td><td></td><td></td><td></td><td></td><td></td><td></td><td></td><td></td><td>x</td><td>x</td><td>x</td><td></td><td></td></tr>
<tr><td>Frozen buffy-coat</td><td></td><td></td><td></td><td></td><td></td><td></td><td></td><td></td><td></td><td></td><td></td><td></td><td></td><td></td><td></td><td></td><td></td><td></td><td>x</td><td>x</td><td>x</td></tr>
<tr><td></td><td>Patient</td><td>P1</td><td></td><td>P2</td><td></td><td></td><td></td><td></td><td></td><td></td><td></td><td></td><td></td><td></td><td></td><td></td><td></td><td></td><td></td><td></td><td></td><td></td></tr>
</table>

Supplementary Materials:

Table S2. Haplotype counts and relative frequencies all two-locus haplotypes in samples C1 and C2 at 9 different PCR conditions. Based on a subset of 4'000 raw Nanopore long-range reads from cDNA *DPYD* amplicon mapped to hg38. "Reads" consist of read sequences that span across all variant positions in the sample. Haplotypes 1-4 assigned in descending order from the overall most to least frequently occurring variant-pair combination. "Other" category comprises of all reads with deletions, insertions, and/or wrong basecalling in at least one of the two variants positions.

			**Haplotype 1**	**Haplotype 2**	**Haplotype 3**	**Haplotype 4**	
	**PCR**	**Reads**	**c.85T/c.1236G**	**c.85C/c.1236A**	**c.85T/c.1236A**	**c.85C/c.1236G**	**Other**
C1	4ng, 30cycles	3168	2145 (67.7%)	730 (23%)	98 (3.1%)	36 (1.1%)	159 (5%)
	4ng, 35cycles	2980	1800 (60.4%)	665 (22.3%)	189 (6.3%)	169 (5.7%)	157 (5.3%)
	4ng, 40cycles	2828	1740 (61.5%)	501 (17.7%)	236 (8.3%)	223 (7.9%)	128 (4.5%)
	8ng, 30cycles	3137	2094 (66.8%)	746 (23.8%)	101 (3.2%)	45 (1.4%)	151 (4.8%)
	8ng, 35cycles	2910	1768 (60.8%)	604 (20.8%)	227 (7.8%)	169 (5.8%)	142 (4.9%)
	8ng, 40cycles	2216	1314 (59.3%)	351 (15.8%)	232 (10.5%)	205 (9.3%)	114 (5.1%)
	16ng, 30cycles	3107	2057 (66.2%)	709 (22.8%)	110 (3.5%)	99 (3.2%)	132 (4.2%)
	16ng, 35cycles	2679	1645 (61.4%)	506 (18.9%)	224 (8.4%)	188 (7%)	116 (4.3%)
	16ng, 40cycles	1762	1023 (58.1%)	273 (15.5%)	191 (10.8%)	179 (10.2%)	96 (5.4%)
	**PCR**	**Reads**	**c.1236G/c.1627G**	**c.1236A/c.1627A**	**c.1236G/c.1627A**	**c.1236A/c.1627G**	**Other**
C1	4ng, 30cycles	3168	2028 (64%)	774 (24.4%)	112 (3.5%)	69 (2.2%)	185 (5.8%)
	4ng, 35cycles	2980	1777 (59.6%)	781 (26.2%)	147 (4.9%)	85 (2.9%)	190 (6.4%)
	4ng, 40cycles	2828	1718 (60.7%)	627 (22.2%)	208 (7.4%)	110 (3.9%)	165 (5.8%)
	8ng, 30cycles	3137	1953 (62.3%)	810 (25.8%)	118 (3.8%)	55 (1.8%)	201 (6.4%)
	8ng, 35cycles	2910	1727 (59.3%)	739 (25.4%)	181 (6.2%)	86 (3%)	177 (6.1%)
	8ng, 40cycles	2216	1295 (58.4%)	473 (21.3%)	180 (8.1%)	110 (5%)	158 (7.1%)
	16ng, 30cycles	3107	1951 (62.8%)	785 (25.3%)	137 (4.4%)	44 (1.4%)	190 (6.1%)
	16ng, 35cycles	2679	1632 (60.9%)	626 (23.4%)	162 (6%)	109 (4.1%)	150 (5.6%)
	16ng, 40cycles	1762	1026 (58.2%)	367 (20.8%)	161 (9.1%)	95 (5.4%)	113 (6.4%)
	**PCR**	**Reads**	**c.1236G/c.2846T**	**c.1236A/c.2846A**	**c.1236G/c.2846A**	**c.1236A/c.2846T**	**Other**
C1	4ng, 30cycles	3168	1973 (62.3%)	778 (24.6%)	84 (2.7%)	63 (2%)	270 (8.5%)
	4ng, 35cycles	2980	1645 (55.2%)	681 (22.9%)	202 (6.8%)	182 (6.1%)	270 (9.1%)
	4ng, 40cycles	2828	1553 (54.9%)	471 (16.7%)	312 (11%)	261 (9.2%)	231 (8.2%)
	8ng, 30cycles	3137	1927 (61.4%)	800 (25.5%)	92 (2.9%)	71 (2.3%)	247 (7.9%)
	8ng, 35cycles	2910	1559 (53.6%)	628 (21.6%)	261 (9%)	199 (6.8%)	263 (9%)
	8ng, 40cycles	2216	1160 (52.3%)	352 (15.9%)	292 (13.2%)	226 (10.2%)	186 (8.4%)
	16ng, 30cycles	3107	1897 (61.1%)	750 (24.1%)	125 (4%)	77 (2.5%)	258 (8.3%)
	16ng, 35cycles	2679	1459 (54.5%)	507 (18.9%)	256 (9.6%)	221 (8.2%)	236 (8.8%)
	16ng, 40cycles	1762	929 (52.7%)	277 (15.7%)	211 (12%)	178 (10.1%)	167 (9.5%)
	**PCR**	Reads	**c.1627G/c.2846T**	**c.1627A/c.2846A**	**c.1627G/c.2846A**	**c.1627A/c.2846T**	**Other**
C1	4ng, 30cycles	3168	1909 (60.3%)	793 (25%)	78 (2.5%)	91 (2.9%)	297 (9.4%)
	4ng, 35cycles	2980	1576 (52.9%)	714 (24%)	164 (5.5%)	212 (7.1%)	314 (10.5%)
	4ng, 40cycles	2828	1476 (52.2%)	538 (19%)	244 (8.6%)	310 (11%)	260 (9.2%)
	8ng, 30cycles	3137	1837 (58.6%)	822 (26.2%)	66 (2.1%)	107 (3.4%)	305 (9.7%)
	8ng, 35cycles	2910	1490 (51.2%)	688 (23.6%)	205 (7%)	242 (8.3%)	285 (9.8%)
	8ng, 40cycles	2216	1103 (49.8%)	399 (18%)	250 (11.3%)	256 (11.6%)	208 (9.4%)
	16ng, 30cycles	3107	1786 (57.5%)	777 (25%)	97 (3.1%)	138 (4.4%)	309 (9.9%)

	PCR	Reads					Other
	16ng, 35cycles	2679	1429 (53.3%)	558 (20.8%)	199 (7.4%)	223 (8.3%)	270 (10.1%)
	16ng, 40cycles	1762	898 (51%)	325 (18.4%)	166 (9.4%)	203 (11.5%)	170 (9.6%)
	**PCR**	**Reads**	**c.85T/c.1627G**	**c.85C/c.1627A**	**c.85T/c.1627A**	**c.85C/c.1627G**	**Other**
C1	4ng, 30cycles	3168	2068 (65.3%)	741 (23.4%)	138 (4.4%)	33 (1%)	188 (5.9%)
	4ng, 35cycles	2980	1677 (56.3%)	642 (21.5%)	272 (9.1%)	185 (6.2%)	204 (6.8%)
	4ng, 40cycles	2828	1576 (55.7%)	482 (17%)	359 (12.7%)	248 (8.8%)	163 (5.8%)
	8ng, 30cycles	3137	1964 (62.6%)	742 (23.7%)	166 (5.3%)	47 (1.5%)	218 (6.9%)
	8ng, 35cycles	2910	1631 (56%)	585 (20.1%)	342 (11.8%)	185 (6.4%)	167 (5.7%)
	8ng, 40cycles	2216	1207 (54.5%)	350 (15.8%)	311 (14%)	213 (9.6%)	135 (6.1%)
	16ng, 30cycles	3107	1913 (61.6%)	718 (23.1%)	194 (6.2%)	91 (2.9%)	191 (6.1%)
	16ng, 35cycles	2679	1529 (57.1%)	477 (17.8%)	305 (11.4%)	212 (7.9%)	156 (5.8%)
	16ng, 40cycles	1762	950 (53.9%)	278 (15.8%)	257 (14.6%)	177 (10%)	100 (5.7%)
	**PCR**	**Reads**	**c.85T/c.2846T**	**c.85C/c.2846A**	**c.85T/c.2846A**	**c.85C/c.2846T**	**Other**
C1	4ng, 30cycles	3168	2010 (63.4%)	746 (23.5%)	112 (3.5%)	25 (0.8%)	275 (8.7%)
	4ng, 35cycles	2980	1580 (53%)	568 (19.1%)	298 (10%)	252 (8.5%)	282 (9.5%)
	4ng, 40cycles	2828	1488 (52.6%)	398 (14.1%)	390 (13.8%)	322 (11.4%)	230 (8.1%)
	8ng, 30cycles	3137	1930 (61.5%)	725 (23.1%)	146 (4.7%)	67 (2.1%)	269 (8.6%)
	8ng, 35cycles	2910	1503 (51.6%)	511 (17.6%)	385 (13.2%)	257 (8.8%)	254 (8.7%)
	8ng, 40cycles	2216	1134 (51.2%)	281 (12.7%)	367 (16.6%)	268 (12.1%)	166 (7.5%)
	16ng, 30cycles	3107	1859 (59.8%)	687 (22.1%)	181 (5.8%)	117 (3.8%)	263 (8.5%)
	16ng, 35cycles	2679	1394 (52%)	403 (15%)	356 (13.3%)	278 (10.4%)	248 (9.3%)
	16ng, 40cycles	1762	899 (51%)	232 (13.2%)	264 (15%)	212 (12%)	155 (8.8%)
			**Haplotype 1**	**Haplotype 2**	**Haplotype 3**	**Haplotype 4**	
	**PCR**	**Reads**	**c.85T/c.1236G**	**c.85C/c.1236A**	**c.85T/c.1236A**	**c.85C/c.1236G**	**Other**
C2	4ng, 30cycles	3101	1577 (50.9%)	1195 (38.5%)	87 (2.8%)	53 (1.7%)	189 (6.1%)
	4ng, 35cycles	2952	1449 (49.1%)	967 (32.8%)	214 (7.2%)	172 (5.8%)	150 (5.1%)
	4ng, 40cycles	1618	773 (47.8%)	449 (27.8%)	160 (9.9%)	147 (9.1%)	89 (5.5%)
	8ng, 30cycles	3120	1694 (54.3%)	1095 (35.1%)	93 (3%)	69 (2.2%)	169 (5.4%)
	8ng, 35cycles	2577	1214 (47.1%)	799 (31%)	248 (9.6%)	181 (7%)	135 (5.2%)
	8ng, 40cycles	861	377 (43.8%)	261 (30.3%)	90 (10.5%)	88 (10.2%)	45 (5.2%)
	16ng, 30cycles	2991	1547 (51.7%)	1044 (34.9%)	123 (4.1%)	97 (3.2%)	180 (6%)
	16ng, 35cycles	2071	987 (47.7%)	590 (28.5%)	206 (9.9%)	179 (8.6%)	109 (5.3%)
	16ng, 40cycles	635	282 (44.4%)	159 (25%)	88 (13.9%)	73 (11.5%)	33 (5.2%)
	**PCR**	**Reads**	**c.496G/c.1236G**	**c.496A/c.1236A**	**c.496G/c.1236A**	**c.496A/c.1236G**	**Other**
C2	4ng, 30cycles	3101	1371 (44.2%)	1255 (40.5%)	56 (1.8%)	245 (7.9%)	174 (5.6%)
	4ng, 35cycles	2952	1276 (43.2%)	1080 (36.6%)	123 (4.2%)	306 (10.4%)	167 (5.7%)
	4ng, 40cycles	1618	691 (42.7%)	504 (31.1%)	106 (6.6%)	213 (13.2%)	104 (6.4%)
	8ng, 30cycles	3120	1465 (47%)	1149 (36.8%)	64 (2.1%)	257 (8.2%)	185 (5.9%)
	8ng, 35cycles	2577	1084 (42.1%)	900 (34.9%)	156 (6.1%)	278 (10.8%)	159 (6.2%)
	8ng, 40cycles	861	339 (39.4%)	304 (35.3%)	60 (7%)	110 (12.8%)	48 (5.6%)
	16ng, 30cycles	2991	1356 (45.3%)	1108 (37%)	88 (2.9%)	243 (8.1%)	196 (6.6%)
	16ng, 35cycles	2071	905 (43.7%)	686 (33.1%)	112 (5.4%)	247 (11.9%)	121 (5.8%)
	16ng, 40cycles	635	267 (42%)	195 (30.7%)	59 (9.3%)	82 (12.9%)	32 (5%)
	**PCR**	**Reads**	**c.85T/c.496G**	**c.85C/c.496A**	**c.85T/c.496A**	**c.85T/c.496G**	**Other**
C2	4ng, 30cycles	3101	1374 (44.3%)	1224 (39.5%)	270 (8.7%)	48 (1.5%)	185 (6%)
	4ng, 35cycles	2952	1315 (44.5%)	1052 (35.6%)	306 (10.4%)	92 (3.1%)	187 (6.3%)

4ng, 40cycles	1618	725 (44.8%)	519 (32.1%)	195 (12.1%)	77 (4.8%)	102 (6.3%)
8ng, 30cycles	3120	1482 (47.5%)	1121 (35.9%)	275 (8.8%)	55 (1.8%)	187 (6%)
8ng, 35cycles	2577	1157 (44.9%)	903 (35%)	262 (10.2%)	78 (3%)	177 (6.9%)
8ng, 40cycles	861	366 (42.5%)	302 (35.1%)	92 (10.7%)	39 (4.5%)	62 (7.2%)
16ng, 30cycles	2991	1372 (45.9%)	1085 (36.3%)	256 (8.6%)	71 (2.4%)	207 (6.9%)
16ng, 35cycles	2071	928 (44.8%)	694 (33.5%)	238 (11.5%)	88 (4.2%)	123 (5.9%)
16ng, 40cycles	635	277 (43.6%)	187 (29.4%)	82 (12.9%)	45 (7.1%)	44 (6.9%)

Supplementary Materials:

Table S3. Multivariable logistic regression of the relationship between PCR-recombination of *DPYD* variants c.85T>C and c.1236G>A in sample C1 and sample C2 and different PCR conditions. Template cDNA input of 4, 8, and 16 ng/μL, and 30, 35, and 40 PCR-amplification cycles were used to make cDNA based full-length *DPYD* amplicons.

Sample	Variants	Coefficients:	Estimate	Std. Error	z value	Pr (>|z|)
C1	c.85-c.1236	(Intercept)	-7.07002	0.194429	-36.363	< 2e-16***
		cDNA input	0.02826	0.004008	7.051	1.77E-12***
		PCR cycles	0.135959	0.005164	26.329	< 2e-16***
C2	c.85-c.1236	(Intercept)	-7.58871	0.220957	-34.345	< 2e-16***
		cDNA input	0.035928	0.004506	7.974	1.54E-15***
		PCR cycles	0.15382	0.005995	25.658	< 2e-16***

Significance codes: $p \leq 0.001$ = ***, $p \leq 0.01$ = **, $p \leq 0.05$ = *

Supplementary Materials:

Table S4. Blood collected in EDTA tubes from individual 19. Buffy-coat was extracted at day 0, day 3, or immediately stored at -80°C for 3 days. RNA isolated from each buffy-coat sample was used to amplify full-length *DPYD* and sequenced with Oxford Nanopore Technologies GridION sequencer. A subset of 4000 raw reads per sample were filtered and used for haplotype phasing and categorized as either "True", "Recombined", or "Other". "Filtered reads" consist of sequences that span across all three variant positions.

**Storage condition**					**Haplotypes (Reads [%])**		
**Temperature**	**Days**	**Reads**	**Filtered reads**	**Two-locus haplotypes**	**True**	**Recombined**	**Other**
Ambient	0	4000 (of 66396)	2465	c.85-c.496	2181 (88.5)	156 (6.3)	128 (5.2)
				c.496-c.1627	2111 (85.6)	197 (8)	157 (6.4)
Ambient	3	4000 (of 50372)	2679	c.85T-c.496	2414 (90.1)	140 (5.2)	125 (4.7)
				c.496-c.1627	2314 (86.4)	202 (7.5)	163 (6.1)
-80°C	3	4000 (of 4219)	3095	c.85-c.496	2583 (83.5)	403 (13)	109 (3.5)
				c.496-c.1627	2434 (78.6)	497 (16.1)	164 (5.3)

Supplementary Materials:

Table S5. List of primers used for PCR. Primers 1-4 used for cDNA based Sanger Sequencing of regions of *DPYD,* flanking the exons in question. Primers 5-6 were used for full length *DPYD* transcript amplification.

No	Primer	Direction	Sequence (5'-3')	Amplicon bp
1	*DPYD* cDNA Ex 3-8	f	GCGGACATCGAGAGTATCCTG	849
2	*DPYD* cDNA Ex 3-8	r	GGCATCTTTATTGGGTTCTGGC	
3	*DPYD* cDNA Ex 13-16	f	GTGATGTCGTTGGTTTGGCT	704
4	*DPYD* cDNA Ex 13-16	r	GACATTTGGGGTCAGCTTGG	
5	*DPYD* cDNA Ex 1-23	f	CGCAAGGAGGGTTTGTCACTG	3’384
6	*DPYD* cDNA Ex 1-23	r	GAACATCCAATTAACTGCCACAC	

Supplementary Materials:

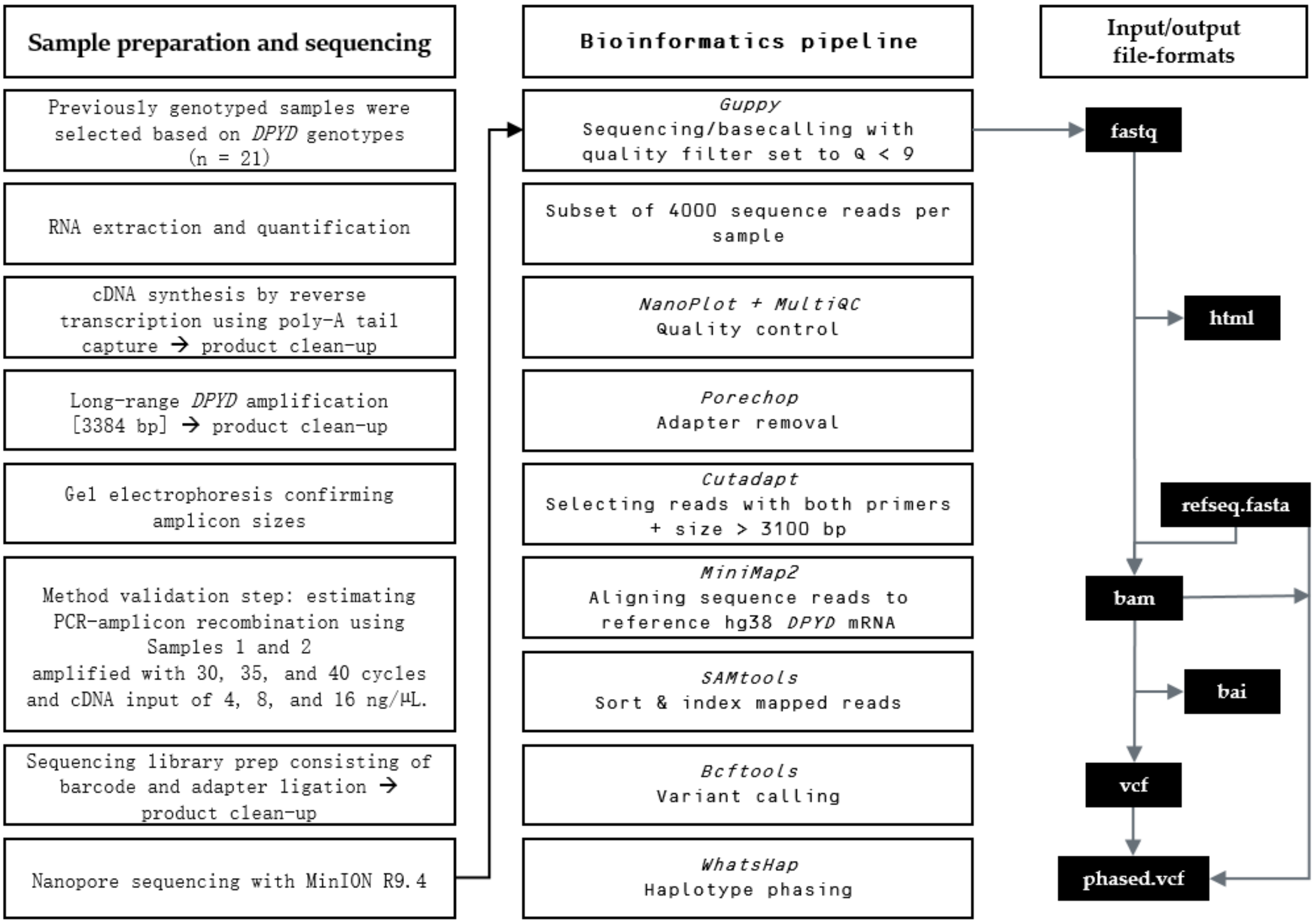

Figure S1. Workflow for phased sequence-based genotyping of dihydropyrimidine dehydrogenase (*DPYD*)
